# Association Between Urinary Metal Levels and Chronic Kidney Dysfunction in Rural China: A Study on Sex-Specific Differences

**DOI:** 10.3390/toxics13010055

**Published:** 2025-01-14

**Authors:** Kaisheng Teng, Qinyi Guan, Qiumei Liu, Xiaoting Mo, Lei Luo, Jiahui Rong, Tiantian Zhang, Wenjia Jin, Linhai Zhao, Songju Wu, Zhiyong Zhang, Jian Qin

**Affiliations:** 1 Department of Environmental and Occupational Health, Guangxi Medical University, Nanning 530021, China; tengks@126.com (K.T.); island0924@163.com (Q.G.); liuqm1029@163.com (Q.L.); mmmoxt@163.com (X.M.); luolei202412@163.com (L.L.); rongjiahui1999@163.com (J.R.); 17878242364@163.com (T.Z.); jxh6636409@163.com (W.J.); zhaolinhai2022@163.com (L.Z.); wsongju@163.com (S.W.); 2 School of Public Health, Guilin Medical University, 20 Lequn Road, Guilin 541001, China; 3 Guangxi Health Commission Key Laboratory of Entire Lifecycle Health and Care, Guilin Medical University, Guilin 541199, China; 4 The Guangxi Key Laboratory of Environmental Exposomics and Entire Lifecycle Heath, Department of Environmental Health and Occupational Medicine, School of Public Health, Guilin Medical University, Zhiyuan Road No.1, Guilin 541199, China; 5 Guangxi Colleges and Universities Key Laboratory of Prevention and Control of Highly Prevalent Diseases, Guangxi Medical University, Nanning 530021, China; 6 Guangxi Key Laboratory of Environment and Health Research, Guangxi Medical University, Nanning 530021, China; 7 Key Laboratory of Longevity and Aging-Related Diseases of Chinese Ministry of Education, Guangxi Medical University, Nanning 530021, China

**Keywords:** urinary metal, chronic kidney disease, quantile g-computation, weighted quantile sum, bayesian kernel machine regression

## Abstract

Background: While current epidemiological studies have documented associations between environmental metals and renal dysfunction, the majority have concentrated on plasma metal levels. The relationship between urinary metal exposure and chronic kidney disease (CKD) remains contentious, particularly within specific demographic groups. Methods: This cross-sectional study included 2919 rural Chinese adults recruited between 2018 and 2019. Urine metals were measured by ICP-MS. Least absolute shrinkage and selection operator (LASSO) regression was employed to identify metals significantly associated with CKD. Then, we used binary logistic regression, along with restricted cubic spline (RCS) models, to assess the individual exposure effects of specific metals on CKD. Quantile g-computation, weighted quantile sum regression, and Bayesian kernel machine regression (BKMR) models were applied to evaluate combined effects of metal exposures on CKD. Gender-stratified analyses were also conducted to explore these associations. Results: LASSO identified seven metals (V, Cu, Rb, Sr, Ba, W, Pb) with significant impacts on CKD. In single-metal models, Cu and W exhibited a positive correlation with CKD, whereas V, Rb, Sr, Ba, and Pb showed significant negative correlations (all *p* < 0.05). RCS analysis revealed nonlinear associations between V, Cu, Ba, Pb, and CKD (all *p*-nonlinear < 0.05). In the multi-metal model, quantile-based g-computation demonstrated a collective negative association with CKD risk for the seven mixed urinary metal exposures (OR (95% CI) = −0.430 (−0.656, −0.204); *p* < 0.001), with V, Rb, Sr, Ba, and Pb contributing to this effect. The WQS model analysis further confirmed this joint negative association (OR (95% CI): −0.885 (−1.083, −0.899); *p* < 0.001), with V as the main contributor. BKMR model analysis indicated an overall negative impact of the metal mixture on CKD risk. Interactions may exist between V and Cu, as well as Cu and Sr and Pb. The female subgroup in the BKMR model demonstrated consistency with the overall association. Conclusions: Our study findings demonstrate a negative association between the urinary metal mixture and CKD risk, particularly notable in females. Joint exposure to multiple urinary metals may involve synergistic or antagonistic interactions influencing renal function. Further research is needed to validate these observations and elucidate underlying mechanisms.

## 1. Introduction

Chronic kidney disease (CKD) affects approximately 1 billion people worldwide, making it a significant public health concern [1]. In the United States, more than 16 million people suffer from moderate CKD, with a growing proportion of older adults affected [2,3]. China has the highest number of CKD patients globally, totaling 132.3 million in 2017 [4]. A five-year cohort study focused on adults aged 55 and older in China indicated that moderate to severe renal impairment increases the risk of cardiovascular events [5]. CKD acts as a risk factor for cardiovascular, metabolic, and psychological disorders, imposing substantial healthcare and economic burdens worldwide [6,7,8,9]. The global aging population exacerbates these challenges, with kidney disease prevalence influencing healthy aging initiatives. Additionally, CKD incidence rates are notably higher among rural residents compared to their urban counterparts in China [10]. Thus, monitoring CKD prevalence among elderly rural populations is crucial in promoting strategies aimed at fostering healthy aging societies.

CKD is a complex condition influenced by both genetic and environmental factors [11]. Research has shown that exposure to environmental metal pollutants, particularly cadmium and arsenic, can accelerate the progression of CKD [12,13]. These metals enter the human body through the air, food, or water, and accumulate in organs such as the liver and kidneys [14,15,16]. While plasma metal concentrations are commonly used as indicators of exposure, urine—being the primary excretory pathway for metal metabolites—provides a more direct reflection of long-term metal exposure and potential kidney damage [17,18]. Compared to plasma metals, studying the relationship between urinary metals and CKD is more meaningful, because changes in urinary metal concentrations (e.g., As, Ba, Ni, U, etc.) may more sensitively reflect the kidney’s response to metal toxicity, thus contributing to the early detection of CKD risk [19]. Therefore, exploring the relationship between urinary metals and CKD can help in the early detection of CKD risks and provide more targeted strategies for its prevention and management.

However, existing research on the relationship between environmental metal exposure and kidney health remains inconsistent. Additionally, while much research has focused on the individual effects of single metals, there is limited understanding of the combined effects of multiple metals. This gap may lead to biased estimates of the effects of individual metals [20]. One study found that lead can competitively replace calcium, resulting in elevated calcium concentrations in the body [21]. Other studies have suggested that copper may either compete with or interact synergistically with metals such as zinc and iron, leading to changes in the concentrations of these metals in the body [22,23]. Furthermore, in daily life, individuals are often exposed to mixtures of multiple metals, and the effects of these metal combinations may differ significantly from the simple additive effects of individual metals. Therefore, investigating the relationship between exposure to metal mixtures and CKD is of great significance for CKD prevention.

Accordingly, we conducted a cross-sectional study targeting middle-aged and elderly populations in rural China, employing various statistical models to evaluate the relationship between exposure to metal mixtures and the risk of CKD. Additionally, we investigated sex-specific differences in this association. The aim of this study is to provide crucial scientific evidence for a better understanding of the relationship between metal exposure and CKD, as well as to offer valuable insights for developing environmental health standards and strategies for CKD prevention in rural areas.

## 2. Materials and Methods

### 2.1. Study Participants

**Figure 1:**
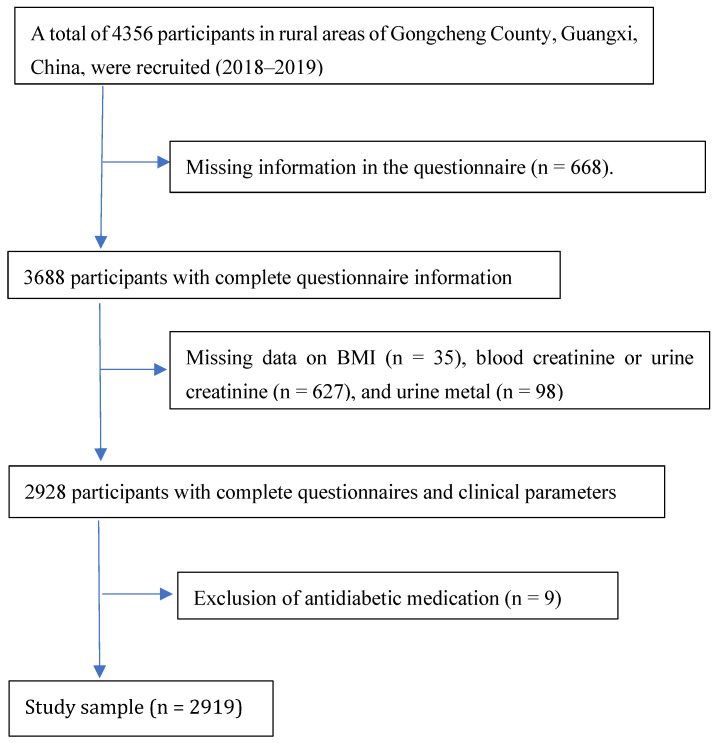
Participant selection and exclusion details.

From 2018 to 2019, a cross-sectional survey was conducted among the general rural population of Gongcheng Yao Autonomous County, situated in southwestern Guangxi, China. Participants meeting the following criteria were included: (A) aged ≥ 30 years; (B) residents of the study area. A total of 4356 adults were initially recruited. Exclusions were based on the following criteria: (A) incomplete questionnaire responses or missing covariate data; (B) absence of essential data such as height, weight, systolic or diastolic blood pressure, blood/urine creatinine, total cholesterol, fasting blood glucose, etc.; (C) missing urinary metal detection data; (D) use of diuretics or other medications. Ultimately, 2919 participants were included in the final analysis (see detailed participant selection and exclusion flowchart in Figure 1). The study protocol was approved by the Ethics Committee of Guilin Medical University (Approval No.: 20180702-3), and informed consent was obtained from all participants.

### 2.2. Urinary Metal Determination

Midstream urine samples were collected and stored at −80 °C until analysis. Prior to analysis, frozen urine samples were thawed and homogenized at room temperature using a gradient method. Subsequently, aliquots of urine (1.0 mL) were transferred to polypropylene tubes and diluted with 9.0 mL of 1% HNO_3_ for acidification overnight at 4 °C. The following morning, metal concentrations in urine were measured using inductively coupled plasma mass spectrometry (ICP-MS; NexION 350X, PerkinElmer, Waltham, MA, USA). The ICP-MS operating parameters for this experiment were as follows: plasma gas flow rate was set to 15–18 L/min, nebulizer gas flow rate was 0.9–1.1 L/min, auxiliary gas flow rate was 1.0–1.2 L/min, and the RF power was 1400 W. The sample flow rate was 0.3–0.6 mL/min, with helium used as the collision/reaction cell gas. The detection mode was set to KED collision mode. Detailed procedures have been previously described [24]. We measured 22 urine metals including titanium (Ti), vanadium (V), chromium (Cr), manganese (Mn), iron (Fe), cobalt (Co), nickel (Ni), copper (Cu), zinc (Zn), arsenic (As), selenium (Se), rubidium (Rb), strontium (Sr), molybdenum (Mo), cadmium (Cd), tin (Sn), antimony (Sb), barium (Ba), tungsten (W), thallium (Tl), lead (Pb), and uranium (U). We established the standard curve using standard solutions at concentrations of 0, 0.05, 0.1, 0.2, 0.5, 1, 2, 5, 10, 50, 100, and 200 ppb, achieving an R^2^ ≥ 0.999 (Figure S1). The internal standards (50 ppb Sc, Y, Rh, and Tb) were utilized for signal correction, with recovery rates consistently maintained at 80–120% (Figure S2). Urinary trace-element control products (Seronorm™ Trace Elements Urine Levels 1 and 2) were measured every 30 samples to ensure quality control, which was deemed satisfactory when the measured values fell within the specified range provided in the instructions. Parallel samples, randomly mixed from 100 samples and diluted by the same factor, were analyzed every 15 samples for consistency and accuracy. Additionally, blank samples were analyzed regularly to monitor contamination. Samples with concentrations below the limit of detection (LOD) were replaced by LOD/√2 [25]. Urine metals were normalized to creatinine using the sarcosine oxidase method with a creatinine assay kit (C011-2-1, Nanjing Jiancheng Bioengineering Institute, China) following the manufacturer’s instructions. Each sample was analyzed in triplicate with a relative standard deviation of less than 5%. Final concentrations of urine metals were expressed as μg/g creatinine.

### 2.3. Kidney Outcomes Measurement

On the same day, blood and urine samples were transported under cold chain conditions to Gongcheng County People’s Hospital for biochemical and urinary biomarker analysis. All blood biochemical parameters were analyzed using a Hitachi 7600-020 chemistry analyzer (Kyoto, Japan), following manufacturer instructions. Urinary biochemical parameters were assessed using a URIT-500B urine analyzer (Guilin, China). Parallel samples were analyzed to minimize measurement errors. The estimated glomerular filtration rate (eGFR) is a key indicator of kidney function, used to estimate the kidney’s ability to filter waste and excess substances from the blood. This study evaluates eGFR using the modification of diet in renal disease (MDRD) formula, which calculates eGFR based on serum creatinine (SCr), age, and gender as follows: for males, eGFR = 175 × SCr^−1.234^ × age^−0.179^; for females, eGFR = 175 × SCr^−1.234^ × age^−0.179^ × 0.79, where SCr is serum creatinine in mg/dL and age is in years. Recent studies indicate that the MDRD equation demonstrates lower mean bias compared to the Cockcroft–Gault and CKD-EPI equations [26]. Additionally, the MDRD equation (75%) is more frequently used than the CKD-EPI equation (52%) for estimating eGFR [27]. CKD is defined as eGFR < 60 mL/min/1.73 m^2^. Urinary albumin, an important marker of kidney function [28], was qualitatively assessed during physical examination. According to definition, a urinary albumin-to-creatinine ratio ≥ 30 mg/g is also indicative of CKD.

### 2.4. Determination of Other Variables

Trained investigators utilized standardized and structured questionnaires to gather sociodemographic data through face-to-face interviews. These characteristics encompassed gender, age (divided into <60 years or ≥60 years), educational attainment (incomplete primary education or middle school and higher), smoking habits (defined as consuming at least one cigarette daily), alcohol consumption (defined as consuming at least 50 g of alcohol per month), and engagement in physical labor, including agricultural activities like cultivation, planting, and weeding. Additionally, following standardized protocols, trained personnel conducted physical examinations that included measurements of height, weight, systolic blood pressure, and diastolic blood pressure. Body mass index (BMI, kg/m^2^) was computed as weight (kg) divided by the square of height (m). Diabetes mellitus was defined as fasting blood glucose levels ≥ 7.0 mmol/L, HbA1c ≥ 6.5%, or based on self-reported physician diagnosis or use of antidiabetic medications (insulin or oral hypoglycemic agents). Hypertension was defined as systolic blood pressure ≥ 140 mmHg, diastolic blood pressure ≥ 90 mmHg, or based on self-reported physician diagnosis or use of antihypertensive medications. Hyperuricemia was defined as uric acid levels > 7 mg/dL in males, and >6 mg/dL in females, or based on self-reported physician diagnosis or use of treatment medications [29].

### 2.5. Statistical Analysis

In the study, demographic characteristics, lifestyle habits, and prevalence of chronic diseases were detailed using mean ± standard deviation and composition ratios. The distribution of urinary metals was described using median (interquartile range, IQR). Basic characteristics were compared using *t*-tests or Mann–Whitney U tests for continuous variables, and chi-square tests for categorical variables. Median and percentiles were used to describe creatinine-corrected urinary metal concentrations. Additionally, differences in urinary metals concentrations between individuals with normal and abnormal kidney function were analyzed using Mann–Whitney U tests. Prior to statistical analysis, metal levels were log10-transformed to reduce skewness.

#### 2.5.1. Single-Exposure Models

This study initially employed Pearson correlation analysis to assess the interrelationships among selected metallic elements. Subsequently, a least absolute shrinkage and selection operator (LASSO) regression model was applied to 22 urinary metals to identify those most significantly associated with CKD. All regression analyses considered covariates previously identified in CKD research as potential contributors to disease onset. Logistic regression models were used to evaluate the relationship between logarithmically transformed urinary metal levels and CKD, with concentrations categorized into quartiles relative to the lowest quartile as reference. Adjustments were made for demographic characteristics, lifestyle habits, chronic diseases, and other relevant variables. Gender-specific subgroup analyses were conducted. Furthermore, RCS analysis was employed to explore potential nonlinear relationships between urinary metals and CKD.

#### 2.5.2. Quantile G-Computation (QGC) and Weighted Quantile Sum (WQS) Regression Model

QGC and WQS are innovative and robust models utilized to investigate the collective impacts of urinary metal mixtures on CKD. The QGC model is specifically designed to analyze the relationship between mixed urinary metal exposure and the risk of CKD, while WQS regression further clarifies the proportional contribution of each metal to this association. In the WQS model, it assumes that the included exposures are linear, additive, and unidirectionally influence the target outcome. In contrast, QGC employs a marginal structural model instead of standard regression, enabling flexible estimation of nonlinear and counterbalancing effects of exposures. This approach provides insights into whether each exposure positively or negatively affects the outcome [30]. This study initially employed QGC to analyze the relationship between metal mixtures and CKD, identifying metals with adverse effects on CKD risk. Subsequently, the identified metals were validated using WQS. In this study, WQS indices were constructed based on quartiles of metals, with exposures set at quartile 4. Estimates from the model represent a quarter-unit increase in the exposure mixture. To enhance result stability, the dataset for WQS regression analysis was split into training (40%) and validation (60%) sets, with bootstrap analysis (n = 1000) employed.

#### 2.5.3. Bayesian Kernel Machine Regression (BKMR) Model

This study employed the BKMR model to assess the overall relationship between jointly exposed urinary metals, selected through LASSO, and CKD. Additionally, it investigated potential interactions among these metals. It examined potential interactions and nonlinear dose-responses between these metals and CKD. Metals were maintained at specific percentiles, with one metal increased to the 90th percentile to assess cumulative effects on kidney function. Additionally, it evaluated the relationship between single-metal exposure and kidney function using exposure-response functions, while other metals were maintained at median levels. Finally, predictive response functions explored the effects of other metals at various percentile levels. Given potential metal interactions, the BKMR analysis employed the Markov chain Monte Carlo algorithm with 50,000 iterations and utilized a hierarchical variable selection method [31].

All analyses in this study were conducted using IBM SPSS Statistics version 27 and R Studio version 4.4.0. Single and mixed-effects analyses were performed using the R packages “rms” (version 6.8.1), “gWQS” (version 3.0.5), and “bkmr” (version 0.2.0). Statistical tests were two-sided, with significance defined as *p* < 0.05.

#### 2.5.4. Sensitivity Analyses

To ensure the robustness of the findings, this study conducted three sensitivity analyses. To address potential influences, we independently reconstructed the model under the following conditions: (a) excluding urine samples with extremely high metal concentrations (defined as samples exceeding three times the 99th percentile for each metal); (b) excluding participants with extreme BMI levels (BMI < 15 or ≥40 kg/m^2^); and (c) repeating the analysis using the chronic kidney disease epidemiology collaboration (CKD-EPI) equation to calculate eGFR.

## 3. Results

### 3.1. Demographic Characteristics

Table 1 summarizes the baseline characteristics of the participants. The study included a total of 2919 participants, consisting of 1070 males (36.66%) and 1849 females (63.34%). There were 379 individuals diagnosed with CKD, accounting for 13.60% of the participants, with an average eGFR of 78.89 ± 22.49 mL/min/1.73 m^2^. The proportion of males and smokers among CKD patients was significantly higher compared to those with normal kidney function (*p* < 0.001), and CKD patients were also older on average (*p* < 0.001). Furthermore, BMI (*p* = 0.003), education level (*p* = 0.014), physical labor, hypertension status, diabetes status, and hyperuricemia status (*p* < 0.001) were all associated with CKD outcomes.

### 3.2. Urinary Metal Concentration

The distribution of characteristic urinary metal elements across different populations is presented in Table S1. Among individuals with CKD, levels of Cu, Zn, and W were significantly elevated compared to those with normal renal function (*p* < 0.05). In contrast, levels of V, Rb, Sr, Ba, Pb, Ti, Mn, Fe, Ni, Sb, Tl, and U were significantly lower in the CKD group than in the normal renal function group (*p* < 0.05). Correlations analyses between metals are depicted in Figure S3. Apart from Ni, W, Sn, and Sb, which exhibited no significant correlations with certain urinary metals in the overall population, correlations were observed among other metals. The strongest correlation was found between As and Mo (r = 0.56, *p* < 0.001), while the weakest was between Ni and U (r = −0.14, *p* < 0.001). In females, the strongest correlation was between As and Mo (r = 0.56, *p* < 0.001), while in males, it was between Sr and Ba (r = 0.60, *p* < 0.001). Notably, a weak correlation between Ni and U persisted among both male (r = −0.18, *p* < 0.001) and female participants (r = −0.18, *p* < 0.001).

### 3.3. Single-Metal Model of Urine Metal and CKD

**Figure 2:**
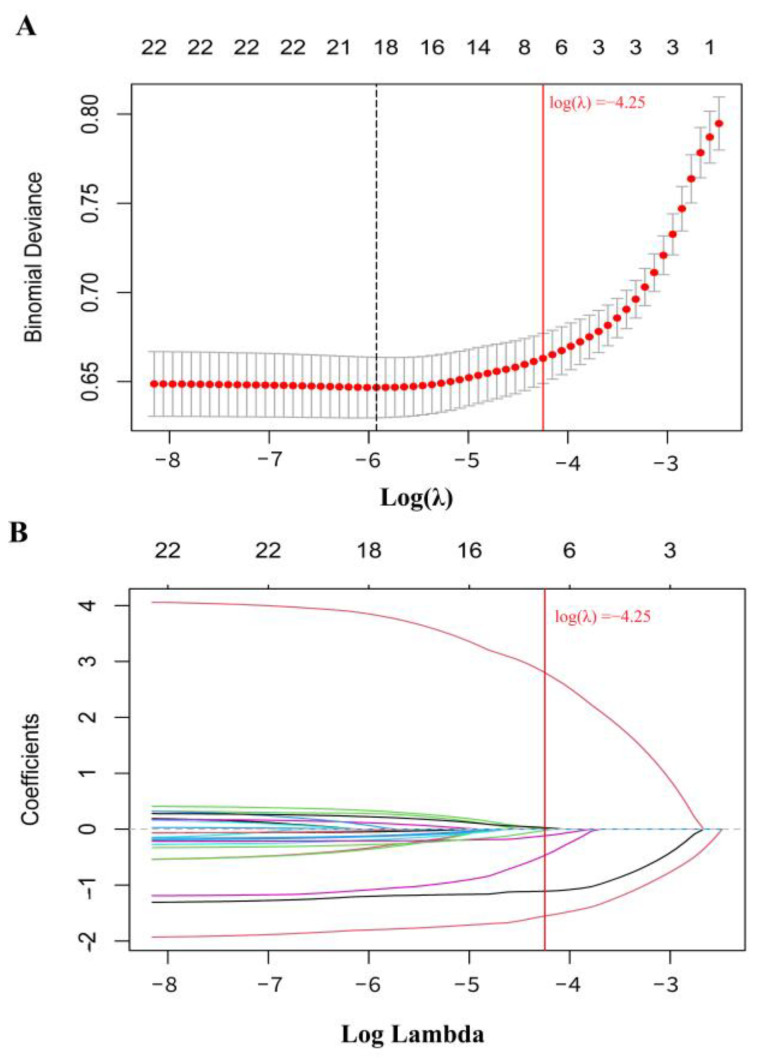
LASSO penalized regression analysis for the associations between 22 urinary metals and the risk of CKD. (**A**) Results from a 10-fold cross-validation of the LASSO model, and (**B**) the β shrinkage process of 22 metal exposures. The solid red line represents the reference line where the binomial deviance was within one standard error of the minimum binomial deviance.

Figure 2 illustrates the relationship between the LASSO penalty parameter (λ) and the shrinkage coefficients during the 10-fold cross-validation process. We defined the penalty parameter (λ = −4.25) as one standard error based on the minimum deviation observed in 10-fold cross-validation (Figure 2A). LASSO stability is achieved at this λ value. Ultimately, the LASSO regression results highlight V, Cu, Rb, Sr, Ba, W, and Pb—seven urinary metals—as significantly associated with CKD (Figure 2B).

**Figure 3:**
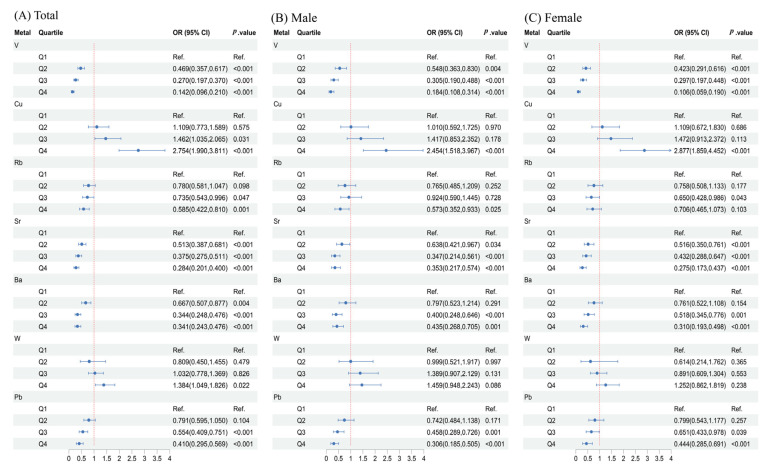
Association between polymetallic exposure and CKD investigated using logistic regression models. Metals identified by LASSO regression were included as predictors in the model. The model was adjusted for and/or sex (male, female), age (<60, ≥60), ethnicity (Han, Yao, other), education (≤6 years, >6 years), smoking status (yes, no), drinking status (yes, no), physical work (yes, no), BMI (<18.5, 18.5–24, ≥24), hypertension (yes, no), diabetes (yes, no), and hyperuricemia (yes, no). Abbreviations: CKD, chronic kidney disease; V, vanadium; Cu, copper; Rb, rubidium; Sr, strontium; Ba, barium; W, tungsten; Pb, lead.

Figure 3 presents the 95% confidence intervals for individual metal exposures assessed using binary logistic regression. In the general population, after adjusting for demographic characteristics, lifestyle habits, and prevalence of chronic diseases, higher levels of Cu (OR [95% CI]: 2.754 [1.990, 3.811]; *p* < 0.001) and W (OR [95% CI]: 1.384 [1.049, 1.826]; *p* = 0.022) continue to show elevated risks of CKD compared to the lowest quartile. Conversely, higher levels of V (OR [95% CI]: 0.142 [0.096, 0.210]; *p* < 0.001), Rb (OR [95% CI]: 0.585 [0.422, 0.810]; *p* = 0.001), Sr (OR [95% CI]: 0.248 [0.201, 0.400]; *p* < 0.001), Ba (OR [95% CI]: 0.341 [0.243, 0.476]; *p* < 0.001), and Pb (OR [95% CI]: 0.410 [0.295, 0.569]; *p* < 0.001) show lower risks of CKD compared to the lowest quartile. Additionally, gender-stratified analysis reveals that while the association between W and CKD diminishes in both male and female participants, Rb (OR [95% CI]: 0.573 [0.352, 0.933]; *p* = 0.025) demonstrates a significant association with CKD in males, whereas in females, a negative association is observed only in the third quartile (OR [95% CI]: 0.650 [0.428, 0.986]; *p* = 0.043).

**Figure 4:**
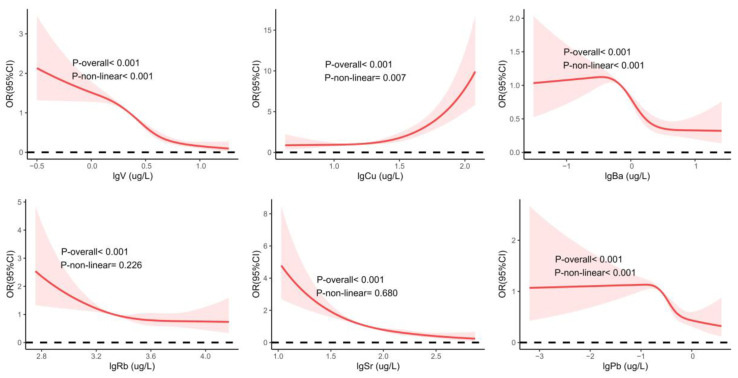
Dose-response relationships of urinary metals and the risk of CKD. RCS regression was employed to analyze the nonlinear relationships between urinary metal levels and CKD risk, adjusting for age, sex, ethnicity, education, drinking and smoking status, BMI, physical work, hypertension, diabetes, and hyperuricemia. Abbreviations: RCS, restricted cubic spline; CKD, chronic kidney disease; V, vanadium; Cu, copper; Rb, rubidium; Sr, strontium; Ba, barium; Pb, lead; lg, log10 transformed.

Figure 4 shows the dose-response relationships between urinary metals and CKD. Nonlinear associations were found for V, Cu, Ba, and Pb with CKD (*p*-overall < 0.001; *p*-nonlinear ≤ 0.001). Additionally, we observed inverse linear associations between Rb (*p*-overall < 0.001; *p*-nonlinear = 0.226) and Sr (*p*-overall < 0.001; *p*-nonlinear = 0.680) and CKD. As urinary levels of Rb and Sr increase, the risk of CKD decreases. Dose-response relationships for other metals with CKD are shown in Figure S4.

### 3.4. Multi-Metals Model of Urine Metal and CKD

#### 3.4.1. QGC Model Assessment of the Association Between Urinary Metals and CKD, Verified by WQS

The QGC model revealed the relationship between urinary metals and CKD. Detailed results are presented in Table 2 and Figure S5. In the overall population, an increase in exposure to all urinary metal mixtures showed a significant inverse association with CKD risk (OR [95%CI] = −0.430 [−0.656, −0.204]; *p* < 0.001). Among the seven urinary metals analyzed, V (43.7%), Pb (17.7%), Ba (15.0%), Sr (14.3%), and Rb (9.3%) appeared to be the primary factors associated with a reduced risk of CKD. In females, this inverse association was also significant (OR [95%CI] = −0.557 [−0.834, −0.280]; *p* < 0.001), with V contributing the most (35.8%). No significant difference was observed in males (OR [95%CI] = −0.394 [−0.883, 0.096]; *p* = 0.115). Subsequently, V, Rb, Sr, Ba, and Pb were included in a WQS regression analysis to assess their combined effect on CKD risk (see Table 2). After adjusting for covariates, consistent with the QGC findings, the combined exposure to these five urinary metals was significantly negatively associated with CKD risk in the overall population (OR [95%CI]: −0.885 [−1.083, −0.899]; *p* < 0.001) and in females (OR [95%CI]: −0.793 [−1.050, −0.536]; *p* < 0.001), with V being the major contributor. Additionally, the WQS model indicated a significant negative correlation between these five combined exposures and CKD risk in males (OR [95%CI]: −0.753 [−1.051, −0.454]; *p* < 0.001), with V as the primary contributor at 49.2%.

#### 3.4.2. BKMR Model Assessment of the Association Between Urinary Metals and CKD

The PIP values obtained from the BKMR model for each metal exposure are summarized in Table S2. Among these, V and Cu exhibited the highest PIP in the overall population (PIP = 1.000). The BKMR model was used to explore potential nonlinear relationships between metals. The results reveal nonlinear associations of V, Cu, Ba, and Pb with CKD risk, findings that were validated even within gender-specific subgroups, as depicted in Figure S6**.** These individual metal dose-response relationships align with the RCS analysis.

**Figure 5:**
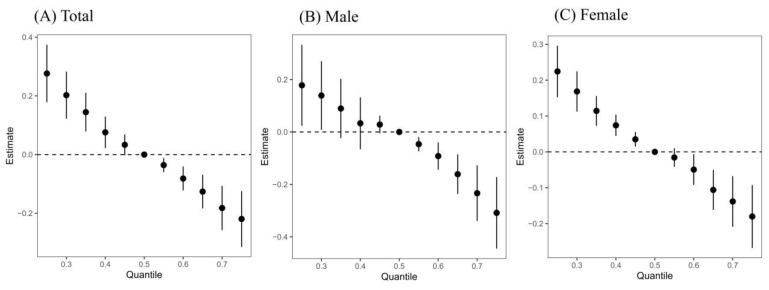
Overall impact (95% CI) of nine metals on CKD when all the metals at particular percentiles were compared to all the metals at their 50th percentile. Data were estimated using the Bayesian kernel machine regression, while adjusting for and/or sex, age, ethnicity, education, drinking and smoking status, BMI, physical work, hypertension, diabetes, and hyperuricemia.

Figure 5 illustrates the cumulative effects of seven metals on CKD risk when simultaneously fixed at different percentiles versus at the median. Our study demonstrates a significant negative correlation overall, indicating that the risk of CKD decreases with higher levels of metal exposure. Subgroup analysis revealed similar results in the females, whereas in males, a significant negative association with CKD risk was observed only when all metals exceeded their P50 values.

**Figure 6:**
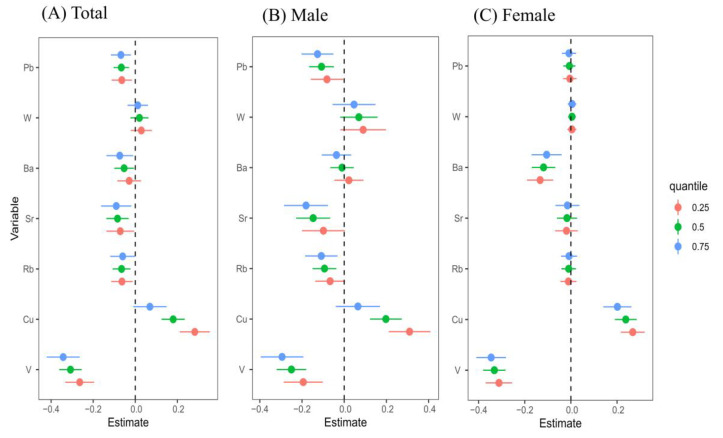
Association (estimate and 95% credible intervals) of each metal increased from the 25th percentile to the 75th percentile with rapid kidney function decline and was observed when other metals in the mixture were fixed at the 25th, 50th, and 75th percentiles. The estimate can be interpreted as the contribution of predictors to the response. Data were estimated using the Bayesian kernel machine regression, while adjusting for and/or sex, age, ethnicity, education, drinking and smoking status, BMI, physical work, hypertension, diabetes, and hyperuricemia. Abbreviations: BKMR, Bayesian kernel machine regression; CKD, chronic kidney disease; V, vanadium; Cu, copper; Rb, rubidium; Sr, strontium; Ba, barium; W, tungsten; Pb, lead.

Figure 6 depicts estimated changes in CKD risk associated with different percentiles (25th, 50th, or 75th) of specific metals compared to their increase from the 25th to the 75th percentile. Vanadium shows a negative association with CKD risk in the overall population (est: −0.264 to −0.343), as well as in males (est: −0.194 to −0.295) and females (est: −0.312 to −0.346). Among the overall population, Pb (est: −0.064 to −0.068), Sr (est: −0.084 to −0.090), and Ba (est: −0.053 to −0.074) are negatively associated with CKD. In males, strontium (est: −0.147 to −0.181), Rb (est: −0.094 to −0.109), and Pb (est: −0.108 to −0.127) show negative associations with CKD. In females, Ba at the 25th percentile (est: −0.133 to −0.105) is negatively associated with CKD. Additionally, lower levels of copper (Cu) show a positive association with CKD risk among female participants at the 25th percentile (est: 0.268 to 0.201), as well as in the overall population (est: 0.283 to 0.180) and in male participants (est: 0.310 to 0.198) when below the 75th percentile.

Figure S7 presents binary exposure-response functions indicating potential interactions between metals. Significant interactions were found between Cu and Ba across the overall population, male, and female subgroups (est = −0.194, *p*-interaction < 0.001), (est = −0.282, *p*-interaction < 0.001), and (est = −0.134, *p*-interaction = 0.009), respectively. For detailed statistics, refer to Table S3.

### 3.5. Sensitivity Analysis Results

We conducted four sensitivity analyses using logistic regression models, and the results are depicted in Figure S8. These sensitivity analyses yielded consistent findings with our primary analysis. When repeating the study after excluding extreme BMI values, urinary metals, and utilizing the CKD-EPI equation for eGFR calculation, elevated CKD risk persisted with high levels of Cu and W compared to the lowest percentile. Conversely, elevated levels of V, Rb, Sr, Ba, and Pb continued to demonstrate lower CKD risk compared to the lowest percentile.

## 4. Discussion

In this study, LASSO regression identified seven urinary metals—V, Cu, Rb, Sr, Ba, W, and Pb—as the most significantly associated with CKD risk. Using logistic regression models, we found significant positive associations between urinary Cu and W levels and CKD risk, while urinary V, Rb, Sr, Ba, and Pb levels showed significant negative associations. Analysis using QGC, WQS, and BKMR models revealed an overall negative combined effect of these metals on CKD risk, with V making the largest contribution. Furthermore, this negative combined effect was more pronounced among female participants. This study highlights the importance of employing diverse statistical methods to assess the correlation between multi-metal co-exposure and CKD. By considering the strengths and limitations of each approach and integrating their findings, we can arrive at more robust conclusions.

Copper (Cu) is an essential trace metal in the body that plays a critical role in renal function. Akhtar et al. demonstrated that copper accumulation in the kidneys of rats can lead to renal dysfunction [32]. Previous epidemiological studies have indicated a significant positive correlation between urinary copper levels and CKD [33,34], consistent with our findings. Our study also observed a nonlinear positive relationship between urinary copper levels and CKD risk, aligning with Yu et al.’s observation of a nonlinear association between urinary copper and eGFR [34], but differing from Guo et al.’s report of a linear positive correlation between blood copper levels and CKD [35]. These discrepancies may arise from variations due to creatinine adjustment of urinary metals or changes in metal levels post-renal metabolism. Despite numerous studies suggesting copper’s nephrotoxic potential, its mechanisms remain elusive. One possible explanation is that elevated copper levels catalyze reactive oxygen species (ROS) formation, generating highly reactive hydroxyl radicals that directly damage cells [36]. Moreover, elevated copper levels can significantly reduce glutathione (GSH) levels and enhance ROS cytotoxicity, thereby increasing copper’s catalytic activity and ROS production, which affects cell function and structure [37]. These findings suggest a detrimental cycle wherein copper ultimately catalyzes high hydroxyl radical production in the kidneys, leading to proximal tubular necrosis [38].

Tungsten (W) is a metal widely used in manufacturing metal alloys and automotive components. Occupational exposure to low levels of tungsten is common due to its broad applications. Exposure typically occurs through water, food, and other pathways, often in combination with exposure to other metals [39,40]. This study identifies a positive correlation between elevated urinary tungsten levels and CKD risk among elderly rural populations in China, consistent with findings by Fox et al. [41], which suggest that increased urine W levels elevate CKD risk. Analysis of NHANES data reveals a linear increase in urinary W levels with rising eGFR [42], ruling out spurious associations due to increased retention of W resulting from declining kidney function. Furthermore, epidemiological research links elevated urinary W levels to T2DM and hypertension [40,43], both of which were associated with CKD risk in our study (see Table 1). Therefore, the association between urinary tungsten levels and CKD risk may be mediated through diabetic nephropathy or hypertensive nephrosclerosis, necessitating further investigation into specific mechanisms. Subgroup analysis indicates that the correlation between tungsten and CKD risk dissipates when examining male or female participants separately. This discrepancy may stem from gender differences or variations in sample structure, underscoring the need for additional research to elucidate these findings.

Vanadium (V) is a naturally occurring metal found in compound forms in the environment, predominantly ingested through dietary sources [44]. Upon absorption, vanadium is transported from the bloodstream to tissues like the liver and kidneys, eventually being excreted via urine [45]. A previous 4.6-year longitudinal study in China among adults demonstrated a significant positive association between plasma vanadium levels and decline in kidney function [46]. In contrast, our study reveals a noteworthy negative correlation between urinary vanadium levels and the risk of CKD, diverging from previous findings. This discrepancy may stem from disparities in urinary versus plasma metal levels in our investigation and could be influenced by reverse causation, where impaired kidney function affects vanadium urinary excretion. Additionally, studies have suggested that vanadium has the potential to modulate the intracellular redox state. This may help alleviate oxidative stress in the body, thereby reducing damage to the renal tubules and glomeruli, and subsequently promoting its excretion at higher concentrations via the kidneys [47]. Another study observed a positive link between urine vanadium and oxidative stress markers associated with metal exposure levels [48]. These findings partially support the hypothesis that oxidative stress contributes to renal damage, thereby influencing the excretion of vanadium in urine. Furthermore, an animal study demonstrated that the concentration of vanadium in the urine of rats exposed to vanadium water was higher than in their plasma, and it correlated positively with the dosage of the contaminant [49]. Another study investigating the pharmacokinetics and oral bioavailability of vanadium sulfate administered via single oral gavage in rats showed an absolute bioavailability ranging from 12.5% to 16.8% [50]. These findings indicate a relatively low absorption rate of vanadium by the body, resulting in higher urinary levels of vanadium compared with blood levels. This disparity may also explain why the association between urinary vanadium and renal function abnormalities differs from that observed with blood vanadium levels and renal function abnormalities.

Barium (Ba) is an alkaline earth metal, and its biological roles or impacts within the human body have not been widely recognized or conclusively determined [51]. A previous cross-sectional study of Chinese adults indicated a negative correlation between urinary Ba levels and renal impairment [52], consistent with our findings. Moreover, research suggests a more pronounced relationship between barium and renal function in females, possibly due to disruptions in estrogen levels. Studies have shown estrogen’s protective role in kidney function [53]. Additionally, studies involving pregnant women and girls aged 8–13 suggest that barium exposure may disrupt reproductive hormone function [54]. Therefore, barium exposure may dysregulate estrogen levels in females, contributing to decreased kidney function. In our study, using the BKMR model to analyze single-metal exposure, we found a negative correlation between urinary Ba levels and CKD risk in female participants, consistent with the aforementioned research findings.

Strontium (Sr) is an alkaline earth metal element considered a trace element in human bones, primarily sourced from dietary intake, especially leafy vegetables, grains, fruits, seafood, and spices [55]. It is predominantly absorbed through the gastrointestinal tract, stored in bones and blood, and excreted via the kidneys [56]. Epidemiological studies have consistently shown a significant positive correlation between plasma Sr levels and declining estimated eGFR, indicative of potential nephrotoxic effects [57]. Clinical research has also highlighted a link between elevated preoperative serum Sr levels and acute kidney injury [58]. Moreover, studies suggest that urinary strontium levels may serve as a protective factor against renal function abnormalities [59]. However, few studies have elucidated the mechanisms by which strontium contributes to renal dysfunction. Current studies indicate that strontium (in the form of soluble salts) acts as an agonist of the calcium-sensing receptor (CaSR), with elevated Sr levels potentially enhancing CaSR expression [60]. Additionally, another study has shown that the expression of CaSR in renal cells can mediate or modulate toxin-induced nephrotoxicity [61]. Therefore, strontium may indirectly alleviate chronic inflammation associated with CKD by influencing calcium metabolism. The precise mechanisms underlying this effect remain to be elucidated through further experimental research.

Our study results reveal a negative correlation between urinary rubidium (Rb) and lead (Pb) levels and CKD in cases of single-metal exposure. However, in combined metal exposure, while the effects of Rb and Pb align with those of the overall metal mixture, their individual contributions appear relatively minor. There appears to be only a modest correlation between Rb and Pb levels in urine and CKD. Research on Rb and kidney function is limited. A previous cross-sectional study in China identified urinary Rb as a protective factor against renal function abnormalities [59]. The study found that rubidium shares similar chemical properties with potassium and can competitively participate in the transport processes of potassium ions across cell membranes, especially in renal tubule cells [62]. In patients with CKD, the renal processing capacity for potassium is diminished, which may result in a reduced excretion of rubidium. Another study found associations between CKD and higher blood lead levels, as well as lower urinary lead levels [63], consistent with our findings. Existing evidence indicates that lead shares certain similarities with calcium and can influence calcium metabolism through competition with calcium [21], potentially contributing to renal dysfunction and exacerbating kidney injury. Consequently, increased lead excretion may help mitigate complications associated with calcium metabolism, thereby impacting the progression of CKD. In reality, people are often simultaneously exposed to multiple metals, which may interact. For example, studies have suggested a combined effect of Pb and Cd in developing CKD [64]. We also observed such interactions among multiple metals.

Additionally, we conducted subgroup analyses by gender to explore potential differences in CKD risk associated with multiple metal exposures. CKD has been extensively studied for gender-based epidemiological differences, particularly in stage G3 (moderate renal dysfunction), where the proportion of female patients is notably higher than that of males [65]. This observation is consistent with our study, in which females represented 52.14% of the CKD patient population. BKMR analysis revealed a significant negative correlation between several urinary metals and CKD in females. This gender-specific association may be attributable to differences in metal accumulation patterns between males and females [66]. A cohort study of workers in the welding and electrical industries found that although male welders are exposed to higher levels of chromium and nickel, female welders have higher concentrations of metals in their urine [67]. Additionally, metal pollutants, such as copper, lead, and barium, can influence metabolic processes by altering hormone levels, particularly estradiol, in females [54,68]. Consequently, these changes may exacerbate kidney damage and accelerate disease progression. Therefore, urinary metals not only affect renal function but also contribute to gender disparities in CKD progression through hormonal modulation, particularly in females. Although current research has not fully elucidated the gender-specific effects of urinary metals on CKD, it is important to give greater attention to the potential impact of gender differences on health, particularly the role of hormones in these differences.

The primary strength of this study lies in its emphasis on the critical role of employing multiple statistical methods to assess the relationship between combined exposure to various metals and renal function impairment. By integrating the strengths and limitations of each method, the study synthesizes results from different approaches to derive robust conclusions. Specifically, we rigorously addressed collinearity and correlation among metal exposures. The LASSO regression method was used to identify metals significantly impacting the risk of rapid decline in kidney function, while the RCS method explored the dose-response relationship between metals and CKD risk. To tackle the multicollinearity inherent in mixed metal exposures, we applied three models—QGC, WQS, and BKMR—ensuring a comprehensive evaluation of the combined impact of multiple metal exposures on renal function impairment. Additionally, the study conducted sensitivity analyses three times to confirm the reliability of our findings.

Despite these strengths, our study has several limitations. Firstly, we only measured total metal concentrations without differentiating between oxidation states and metabolites. Secondly, metals were obtained from a single urine sample, and temporal variations in metal exposure levels were not considered. Lastly, owing to the cross-sectional design of our study, a causal relationship between metal exposure and CKD cannot be inferred as it would be with longitudinal data. Therefore, additional research with longitudinal data is necessary to further investigate and address this issue.

## 5. Conclusions

The results of this study revealed V, Rb, Sr, Ba, and Pb exhibited significant negative correlations with CKD in a single metal model. Conversely, significant positive correlations were found between Cu and W with CKD. RCS analysis demonstrated nonlinear dose-response relationships between urine V, Cu, Ba, Pb, and CKD, while linear dose-response relationships were observed between Rb, Sr, and CKD. Mixture chemical analysis indicated a noteworthy association between exposure to a mixture of seven urinary metals and reduced CKD risk, with V being the primary contributing metal to this negative association, particularly among the female population. Moreover, interactions among multiple urinary metals were identified. Our findings provide crucial scientific evidence for exploring the relationship between urinary exposure to mixed metals and renal dysfunction, laying the groundwork for the development of environmental health standards and strategies for the prevention of chronic kidney disease (CKD) in the future. Further cohort studies involving larger populations are essential to validate these findings, and a detailed elucidation of their underlying mechanisms is crucial.

## Figures and Tables

**Table 1:** Basic characteristics of the study population (n = 2919).

Variables	Total	Chronic Kidney Disease		*p* Value
		Yes	No	
Subjects, n (%)	2919	397 (13.60)	2522 (86.40)	
Sex, n (%)				<0.001
Man	1070 (36.66)	190 (47.86)	880 (34.89)	
Woman	1849 (63.34)	207 (52.14)	1642 (65.11)	
Age, n (%)				<0.001
<60	1490 (51.04)	149 (37.53)	1341 (53.17)	
≥60	1429 (48.96)	248 (62.47)	1181 (46.83)	
BMI, n (%)				0.003
<18.5	224 (7.67)	41 (10.33)	183 (7.26)	
18.5–24	1764 (60.43)	210 (52.90)	1554 (61.62)	
≥24	931 (31.89)	146 (36.77)	785 (31.12)	
Ethnicity, n (%)				0.243
Han	579 (19.84)	91 (22.93)	488 (19.35)	
Yao	2194 (75.16)	288 (72.54)	1906 (75.57)	
Other	146 (5.00)	18 (4.53)	128 (5.08)	
Smoking status, n (%)				<0.001
Yes	518 (17.75)	94 (23.68)	424 (16.81)	
No	2401 (82.25)	303 (76.32)	2098 (83.19)	
Drinking status, n (%)				0.718
Yes	911 (31.21)	127 (31.99)	784 (31.09)	
No	2008 (68.79)	270 (68.01)	1738 (68.91)	
Education level, n (%)				0.014
≤6 years	1846 (63.24)	273 (68.77)	1573 (62.37)	
>6 years	1073 (36.76)	124 (31.23)	949 (37.63)	
Physical work, n (%)				<0.001
Yes	1026 (35.15)	217 (54.66)	1676 (66.46)	
No	1893 (64.85)	180 (45.34)	846 (33.54)	
Hypertension, n (%)				<0.001
Yes	1300 (44.54)	94 (39.66)	1063 (42.15)	
No	1619 (55.46)	143 (60.34)	1459 (57.85)	
Diabetes, n (%)				<0.001
Yes	337 (11.55)	237 (59.70)	260 (10.31)	
No	2582 (88.45)	160 (40.30)	2262 (89.69)	
Hyperuricemia, n (%)				<0.001
Yes	478 (16.38)	117 (29.47)	361 (14.31)	
No	2441 (83.62)	280 (70.53)	2161 (85.69)	
eGFR, mL/min/1.73 m^2^	91.64 ± 19.35	78.89 ± 22.49	93.63 ± 18.03	
UACR, mg/g, n (%)				
≥30	347 (11.89)	347 (87.41)	0 (0.00)	
<30	2572 (88.11)	50 (12.59)	2522 (100.00)	

BMI: body mass index; eGFR: estimated glomerular filtration rate; UACR: the urinary albumin/creatinine ratio. Continuous values are presented as mean ± SD according to their distribution and category variables are shown as numbers (percentage). *p* values of continuous variables were calculated with Student’s *t*-test or Mann–Whitney U test according to their distribution, and those for category variables were calculated with chi-square tests.

**Table 2:** QGC model assessment of the association between urinary metals and chronic kidney disease, verified by WQS.

Variable	V	Cu	Rb	Sr	Ba	W	Pb	OR (95% CI)	*p*-Value
QGC									
Total	−0.448	0.768	−0.107	−0.119	−0.164	0.232	−0.162	−0.430 (−0.656, −0.204)	<0.001
Male	−0.588	0.656	−0.061	−0.064	−0.139	0.344	0.149	−0.394 (−0.883, 0.096)	0.115
Female	−0.358	0.854	−0.145	−0.174	−0.170	0.146	−0.152	−0.557 (−0.834, −0.280)	<0.001
WQS									
Total	−0.508	-	−0.028	−0.189	−0.213	-	−0.061	−0.885 (−1.083, −0.899)	<0.001
Male	−0.492	-	−0.108	−0.093	−0.035	-	−0.271	−0.753 (−1.051, −0.454)	<0.001
Female	−0.574	-	−0.014	−0.147	−0.167	-	−0.098	−0.793 (−1.050, −0.536)	<0.001

Abbreviations: QGC, quantile g-computation; WQS, weighted quantile sum; V, vanadium; Cu, copper; Rb, rubidium; Sr, strontium; Ba, barium; W, tungsten; Pb, lead.

## Data Availability

The data that support the findings of this study are available on request from the corresponding authors, Zhiyong Zhang and Jian Qin, upon reasonable request.

## Supplementary Materials

The following supporting information can be downloaded at https://www.mdpi.com/article/10.3390/toxics13010055/s1, Figure S1: The R^2^ values of the standard curves for the 22 urinary metals; Figure S2: Range of standard recovery rates; Figure S3: Spearman correlations among the mixed exposure of 22 metals in the participants; Figure S4: Dose-response Relationships of Urinary Metals and the Risk of CKD; Figure S5: QGC and WQS regression model weights of each metal contributing to the overall effect; Figure S6: Univariate exposure-response functions and 95% credible intervals (shaded areas) for each metal with the other metals kept at the median; Figure S7: Bivariate exposure-response functions for each metal presented on the upper coordinate axis when the other metal is presented on the right longitudinal axis, holding at different quantiles (25th, 50th, and 75th percentiles), while the other three metals are held at the median; Figure S8: The results of sensitivity analyses; Table S1: The distribution of characteristic urinary metal elements; Table S2: PIP values of metal exposure obtained by BKMR model; Table S3: Metal interaction of the study population.
