# Supplementary material for: Association Between Urinary Metal Levels and Chronic Kidney Dysfunction in Rural China: A Study on Sex-Specific Differences
Source: Toxics. 2025 Jan 14;13(1):55. doi: 10.3390/toxics13010055 (PMC11768882; doi:10.3390/toxics13010055)
Supplement: Supplementary file 1 [file toxics-13-00055-s001.zip › toxics-3404776-supplementary.pdf]

Table. S1 The distribution of characteristic urinary metal elements (n = 2919)

Urinary metals (μg/L)	Total	Chronic kidney disease		*P*-value
		Yes	No	
Vanadium, V	3.60(2.25,5.60)	2.26(1.40,3.59)	3.83(2.43,5.93)	<0.001
Copper, Cu	20.06(14.39,27.90)	24.76(16.72,40.12)	19.47(14.04,26.72)	<0.001
Rubidium, Rb	2874.63(2129.86,3966.52)	2691.05(1951.31,3548.85)	2918.42(2168.30,4015.59)	<0.001
Strontium, Sr	114.66(71.14,174.38)	77.13(43.33,133.34)	119.81(76.82,181.99)	<0.001
Barium, Ba	1.32(0.70,2.46)	0.87(0.47,1.62)	1.41(0.76,2.60)	<0.001
Tungsten, W	0.01(0.00,0.11)	0.03(0.00,0.21)	0.01(0.00,0.10)	<0.001
Lead, Pb	0.38(0.25,0.59)	0.32(0.20,0.48)	0.39(0.26,0.60)	<0.001
Titanium, Ti	467.86(329.68,648.02)	425.52(301.25,575.31)	476.76(332.29,659.77)	<0.001
Chromium, Cr	0.47(0.25,0.47)	0.46(0.28,0.67)	0.47(0.25,0.78)	0.456
Manganese, Mn	0.62(0.25,1.44)	0.53(0.21,1.15)	0.64(0.27,1.49)	0.002
Ferrum, Fe	56.93(36.46,86.27)	51.16(32.04,78.66)	57.84(37.32,87.06)	0.004
Cobalt, Co	0.34(0.17,0.66)	0.33(0.20,0.60)	0.34(0.17,0.65)	0.838
Nickel, Ni	3.08(1.33,5.50)	2.98(1.31,5.25)	3.10(1.32,5.56)	0.283
Zinc, Zn	587.81(407.71,821.70)	669.31(462.02,940.56)	574.95(395.77,805.03)	<0.001
Arsenic, As	41.50(31.04,56.33)	42.10(31.21,59.75)	41.45(30.99,56.09)	0.456
Selenium, Se	28.01(21.52,36.34)	27.54(20.88,35.67)	28.14(21.59,36.39)	0.315
Molybdenum, Mo	65.06(43.74,95.58)	63.25(42.00,95.73)	65.25(44.22,95.58)	0.347
Cadmium, Cd	2.35(1.42,3.79)	2.34(1.42,4.06)	2.35(1.42,3.76)	0.527
Stannum, Sn	0.01(0.01,0.09)	0.01(0.01,0.09)	0.01(0.01,0.09)	0.280
Antimony, Sb	0.00(0.00,0.04)	0.00(0.00,0.03)	0.00(0.00,0.04)	0.016
Thallium, Tl	0.32(0.21,0.49)	0.26(0.16,0.40)	0.33(0.22,0.50)	<0.001
Uranium, U	0.00(0.00,0.02)	0.00(0.00,0.01)	0.00(0.00,0.02)	<0.001

Metals were presented as median (IQR) according to their distribution.

A log10 (lg) conversion is performed on the level of the metal before statistical analysis. *P* values of the metal were calculated using Student’s Mann-Whitney U test, according to their distribution.

Table S2 PIP values of metal exposure obtained by BKMR model

Urinary metals (μg/L)	PIP		
	All	Male	Female
Vanadium, V	1.000	1.000	1.000
Copper, Cu	1.000	1.000	1.000
Rubidium, Rb	0.874	0.879	0.041
Strontium, Sr	0.959	0.960	0.113
Barium, Ba	0.817	0.462	1.000
Tungsten, W	0.509	0.548	0.043
Lead, Pb	0.951	0.906	0.089

Table. S3 Metal interaction of the study population.

Metal interaction		Estimate	value	*P*-interaction
Total				
	V * Cu	-0.1780	-2.95	0.003
	V *Ba	0.117	3.19	0.002
	Cu* Sr	-0.272	-4.45	< 0.001
	Cu* Pb	-0.133	-2.79	0.005
	Cu* Ba	-0.194	-4.39	< 0.001
Male				
	Cu* Pb	-0.253	-2.35	0.019
	Cu* Ba	-0.282	-3.34	< 0.001
	Ba*W	-0.066	-2.85	0.005
Female				
	V * Cu	-0.168	-2.16	0.031
	V * Ba	0.154	3.13	0.002
	Cu* Ba	-0.134	-2.62	0.009

Possible interactions were confirmed using a traditional linear mixed-effects regression model.

Age, sex (male, female), ethnicity (Yao, other), education level (Primary school or below, Middle school or beyond), BMI, smoking status (Current/Former smoker, Never smoker), drinking status (Current/Former drinker, Never drinker), hypertension (yes, no), diabetes (yes, no), hyperuricemia (yes, no) were adjusted as covariates.

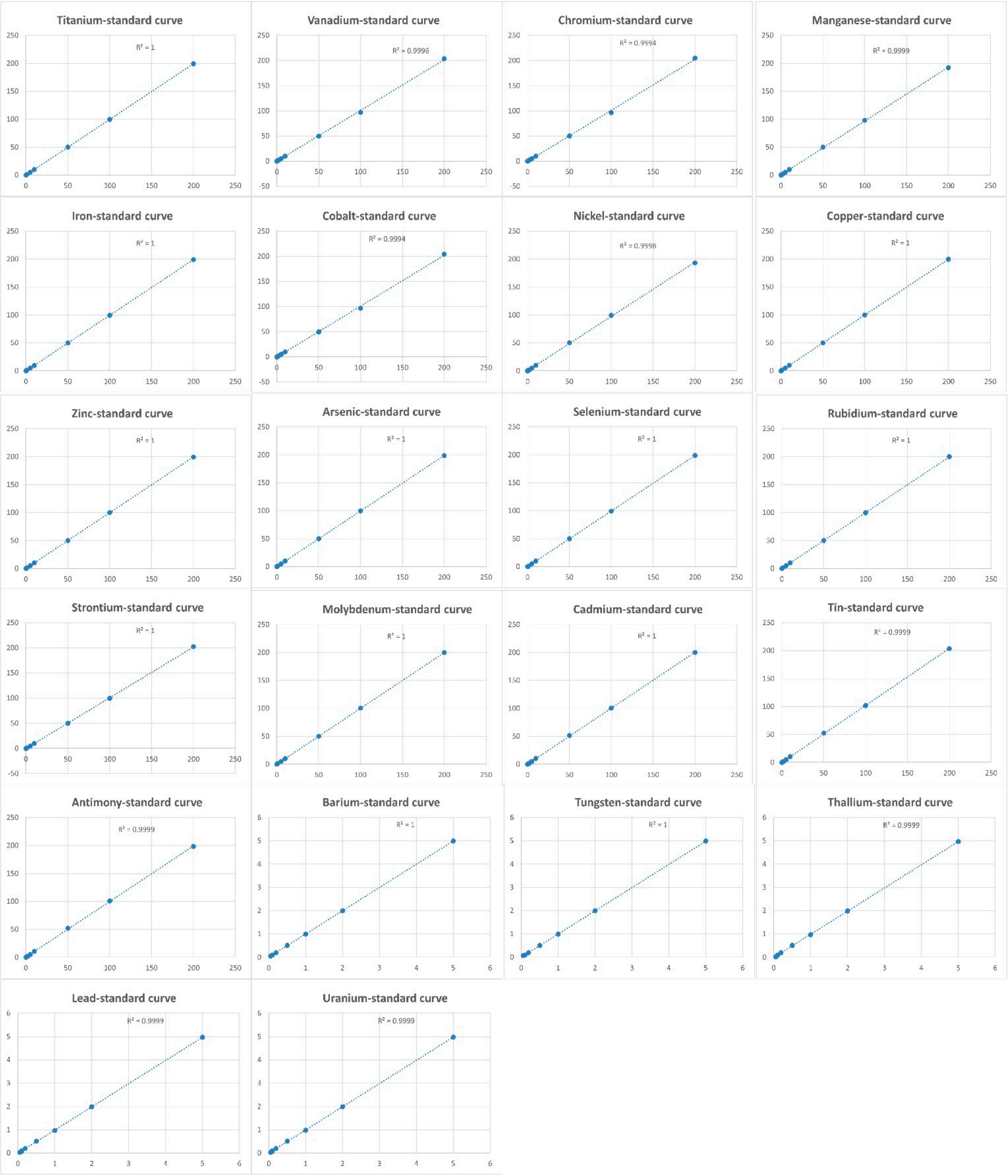

Fig. S1 The R^2 values of the standard curves for the 22 urinary metals.

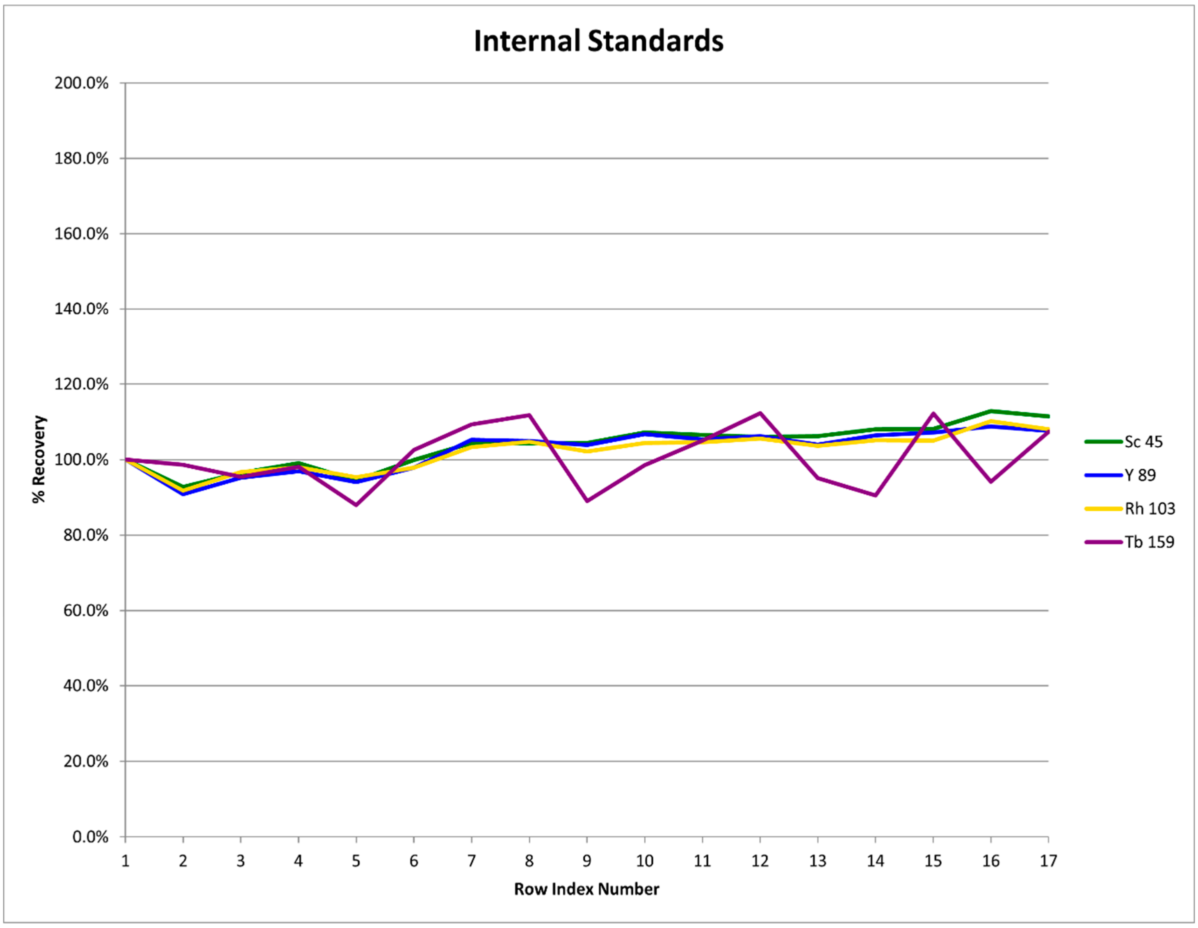

Fig S2. Range of standard recovery rates.

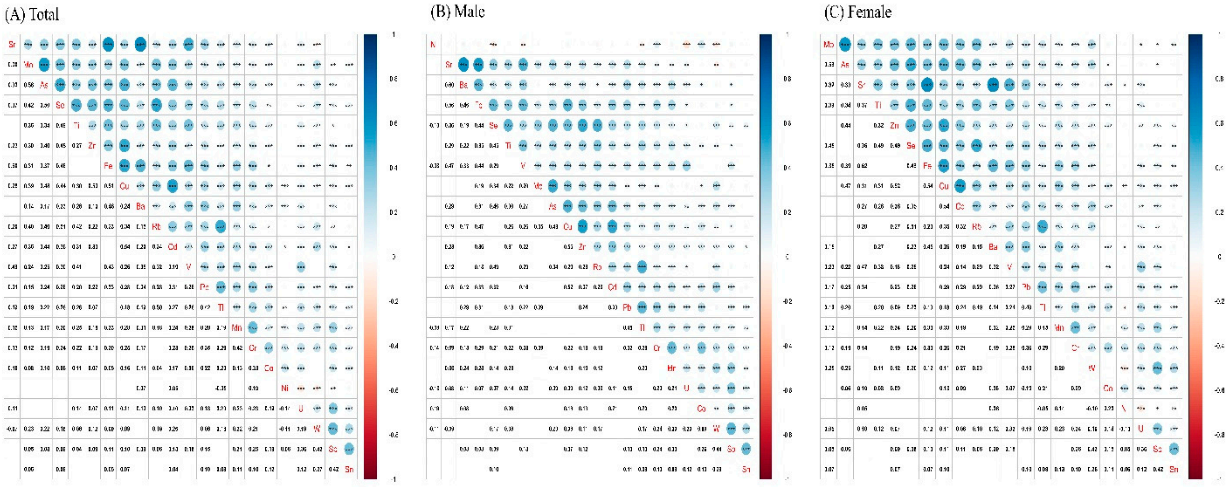

Fig S3. Spearman correlations among the mixed exposure of 22 metals in the participants. The numbers in the lower left corner represent the correlation coefficient, and red denotes the positive correlation, while blue denotes the negative correlation. The darker the color, the greater the correlation coefficient. Abbreviations: V, Vanadium; Cu, Copper; Rb, Rubidium; Sr, Strontium; Ba, Barium; W, Tungsten; Pb, Lead.

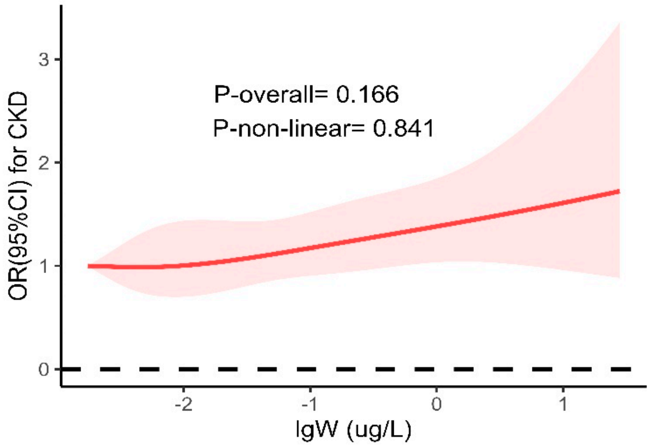

Fig S4. Dose-response Relationships of Urinary Metals and the Risk of CKD. RCS regression was employed to analyze the nonlinear relationships between urinary metal levels and CKD risk, adjusting for age, sex, ethnicity, education, drinking and smoking status, BMI, physical work, hypertension, diabetes, and hyperuricemia. Abbreviations: RCS, restricted cubic spline; CKD, chronic kidney disease; W, Tungsten; lg, log10 transformed.

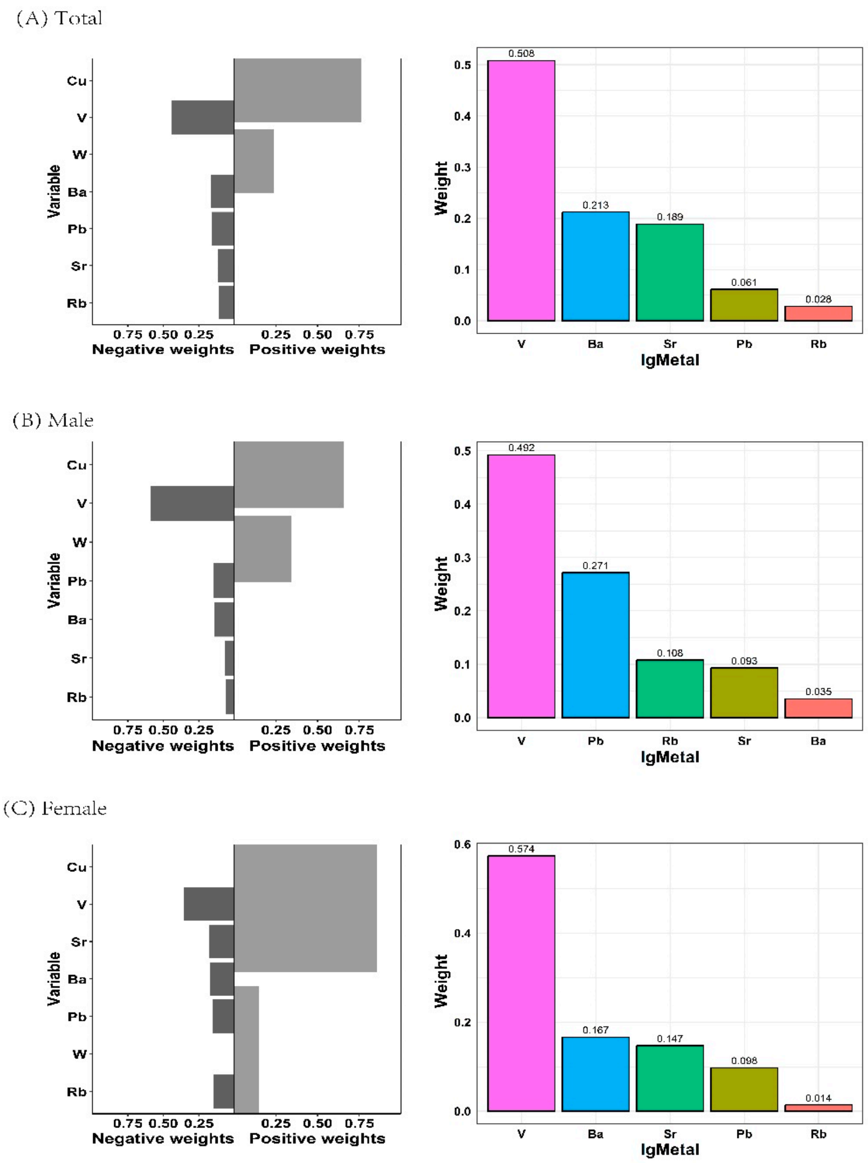

Fig S5. QGC and WQS regression model weights of each metal contributing to the overall effect. Age, sex, ethnicity, education, drinking and smoking status, BMI, physical work, hypertension, diabetes, and hyperuricemia. were considered as covariates. Abbreviations: QGC, quantile g-computation; WQS, weighted quantile sum; V, Vanadium; Cu, Copper; Rb, Rubidium; Sr, Strontium; Ba, Barium; W, Tungsten; Pb, Lead; lg, log10 transformed.

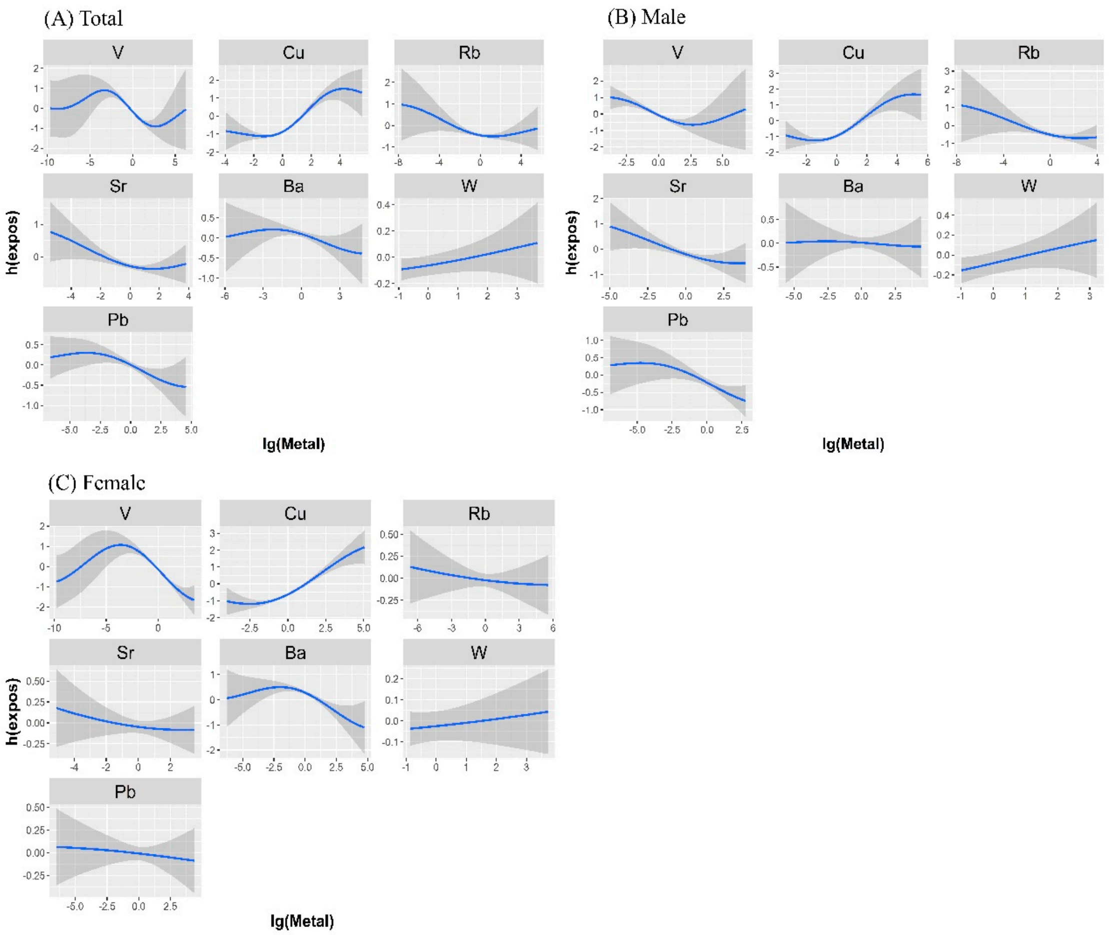

Fig S6. Univariate exposure-response functions and 95% credible intervals (shaded areas) for each metal with the other metals kept at the median. The estimate can be interpreted as the contribution of predictors to the response. Data were estimated using the Bayesian kernel machine regression, while adjusting for and/or sex, age, ethnicity, education, drinking and smoking status, BMI, physical work, hypertension, diabetes, and hyperuricemia. Abbreviations: BKMR, Bayesian kernel machine regression; CKD, chronic kidney disease; V, Vanadium; Cu, Copper; Rb, Rubidium; Sr, Strontium; Ba, Barium; W, Tungsten; Pb, Lead; lg, log10 transformed.

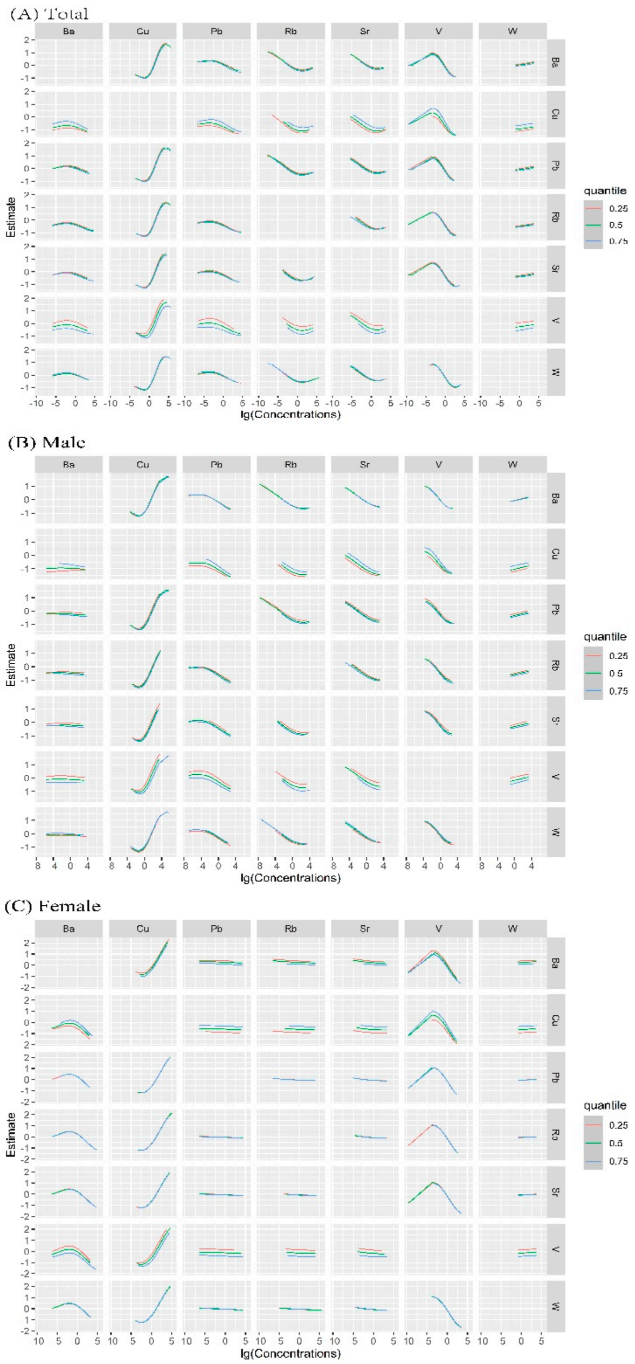

Fig S7. Bivariate exposure-response functions for each metal presented on the upper coordinate axis when the other metal is presented on the right longitudinal axis, holding at different quantiles (25th, 50th, and 75th percentiles), while the other three metals are held at the median. The estimate can be interpreted as the contribution of predictors to the response. Data were estimated using the Bayesian kernel machine regression, while adjusting for and/or sex, age, ethnicity, education, drinking and smoking status, BMI, physical work, hypertension, diabetes, and hyperuricemia. Abbreviations: BKMR, Bayesian kernel machine regression; CKD, chronic kidney disease; V, Vanadium; Cu, Copper; Rb, Rubidium; Sr, Strontium; Ba, Barium; W, Tungsten; Pb, Lead; lg, log10 transformed.

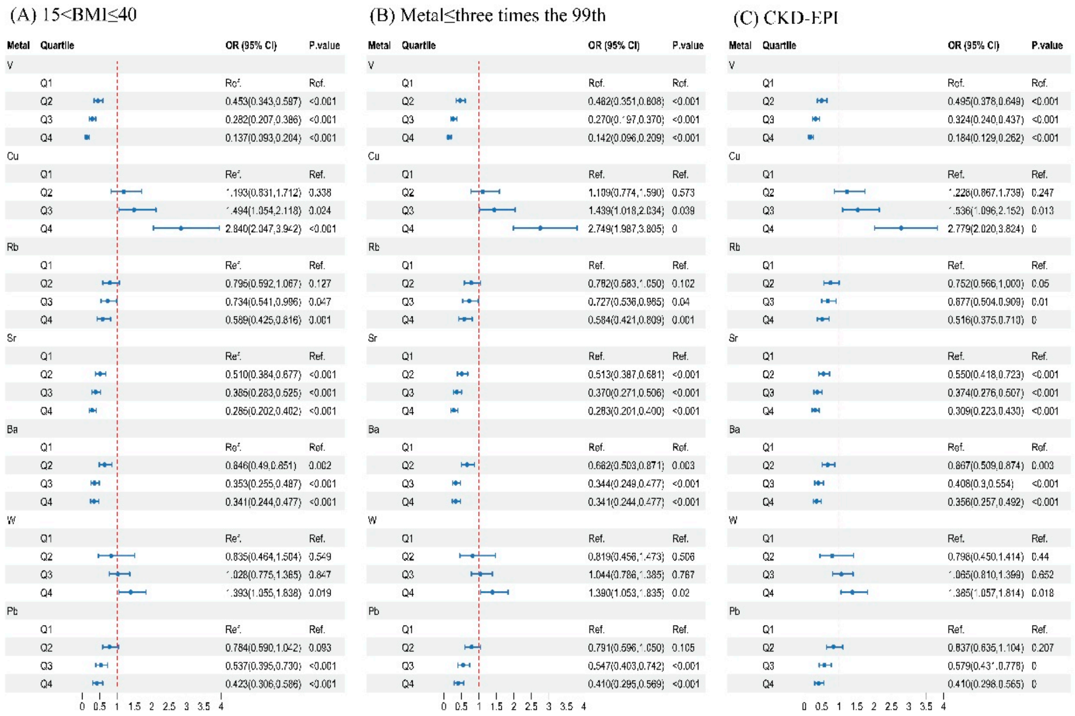

Fig S8. The results of sensitivity analyses. (A) exclude extreme BMI levels; (B) exclude extreme metal levels; (C) using the Chronic Kidney Disease Epidemiology Collaboration (CKD-EPI) equation to calculate eGFR. Data were estimated using logistic Regression Models, while adjusting for sex, age, ethnicity, education, drinking and smoking status, BMI, physical work, hypertension, diabetes, and hyperuricemia. Abbreviations: CKD, chronic kidney disease; V, Vanadium; Cu, Copper; Rb, Rubidium; Sr, Strontium; Ba, Barium; W, Tungsten; Pb, Lead.
